# Supplementary material for: RASSF1A is required for the maintenance of nuclear actin levels
Source: EMBO J. 2019 Jun 7;38(16):e101168. doi: 10.15252/embj.2018101168 (PMC6694222; doi:10.15252/embj.2018101168)
Supplement: Supplementary file 1 — Expanded View Figures PDF [file EMBJ-38-e101168-s001.pdf]

Expanded View Figures

Figure EV1. NE localisation of RASSF1A does not dependent on ATR/ATM kinases.

A Assessment of RASSF1A protein levels in HeLa cells transfected with siRASSF1A. Error bars derive from two independent experiments and represent the SEM.
B Representative confocal images of RASSF1A and Lamin A/C in siRASSF1A-transfected HeLa cells. DNA was stained with DAPI. Scale bars = 10 μm.
C Immunofluorescence images of RASSF1A in MDA-MB-231 cells after treatment with DMSO or 5′-aza-dC demethylating reagent. DNA was stained with DAPI. Lower blot shows the expression levels of RASSF1A levels following treatment. Scale bars = 10 μm.
D Digitonin-permeabilised HeLa cells stained with RASSF1A and the cytoplasmic α-tubulin as a marker of plasma membrane permeabilisation. DNA was stained with DAPI. Scale bars = 10 μm.
E Immunofluorescence detection of phosphorylated RASSF1A (Ser 131). Fluorescence intensity profile of Lamin A/C (red) and pRASSF1A (Ser 131) (green) signals across the HeLa nuclei. Position of line scan indicated by the dashed white line. Scale bars = 10 μm.
F Immunofluorescence images of RASSF1A in HeLa cells treated with DMSO, ATR inhibitor VE821, ATM inhibitor KU5933 and the combination of both. Western blot (bottom) of RASSF1A protein levels following the corresponding treatments. Scale bars = 10 μm.

Data information: Two-tailed Student's *t*-test was used for statistical analysis. ***$P < 0.001$.
Source data are available online for this figure.

 © 2019 The Authors

16 of 15 *The EMBO Journal* e101168 | 2019 © 2019 The Authors

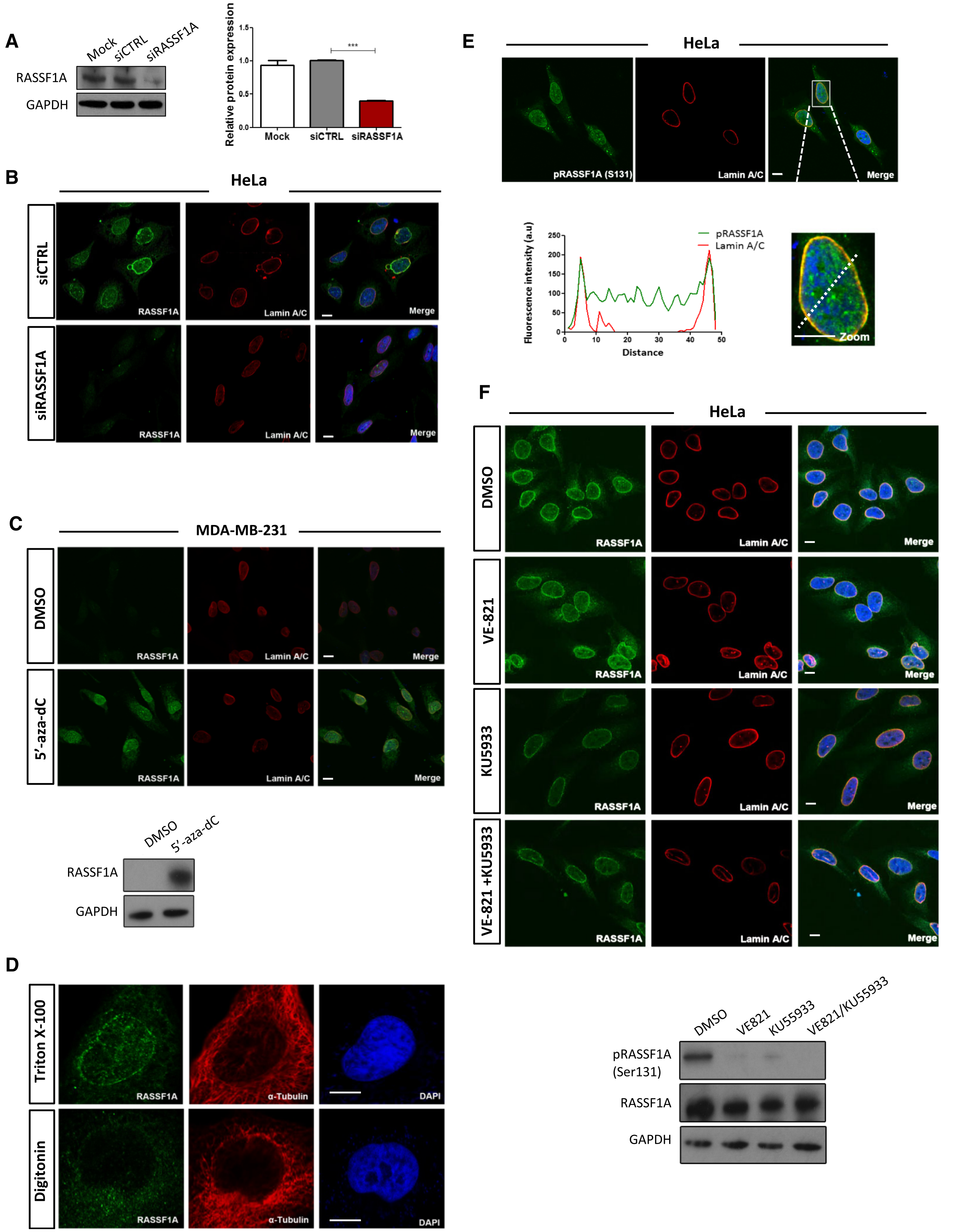
A
Mock
siCTRL
siRASSF1A
RASSF1A
GAPDH
Relative protein expression
***
Mock
siCTRL
siRASSF1A
B
HeLa
siCTRL
siRASSF1A
RASSF1A
Lamin A/C
Merge
C
MDA-MB-231
DMSO
5'-aza-dC
D
Triton X-100
Digitonin
α-Tubulin
DAPI
E
HeLa
pRASSF1A (S131)
Fluorescence intensity (a.u)
Distance
Zoom
F
DMSO
VE-821
KU5933
VE-821 +KU5933
VE821
KU55933
VE821/KU55933
pRASSF1A
(Ser131)

Figure EV1.

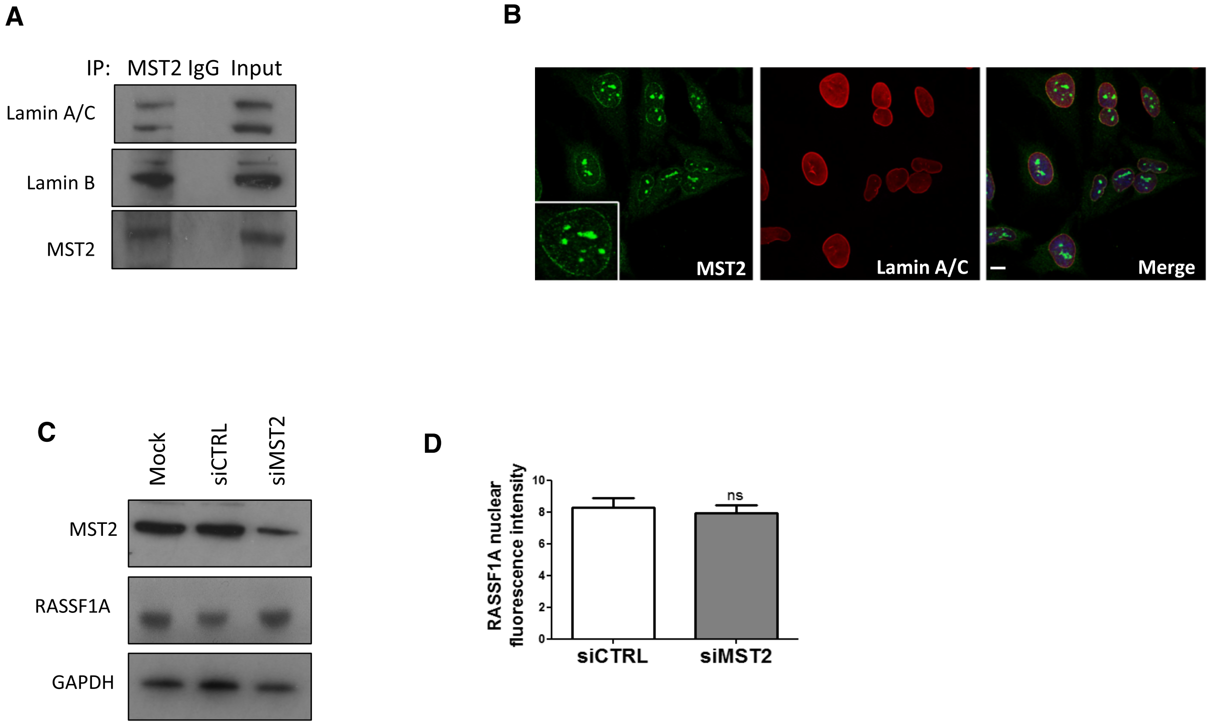

Figure EV2. MST2 binds to Lamins A/C and B.

A Co-immunoprecipitation of endogenous MST2 with Lamin B and Lamin A/C.
B Immunofluorescence images of RASSF1A and MST2 (ab52641). Scale bars = 10 μm.
C Western blot analysis of MST2 and RASSF1A protein levels in siMST2-transfected cells.
D Quantification of nuclear fluorescence intensity of RASSF1A in siMST2-transfected cells. Fluorescence intensity was quantified in 50–100 cells. Error bars derive from three independent experiments and represent the SEM.

Data information: Two-tailed Student's *t*-test was used for statistical analysis.

Source data are available online for this figure.

 © 2019 The Authors

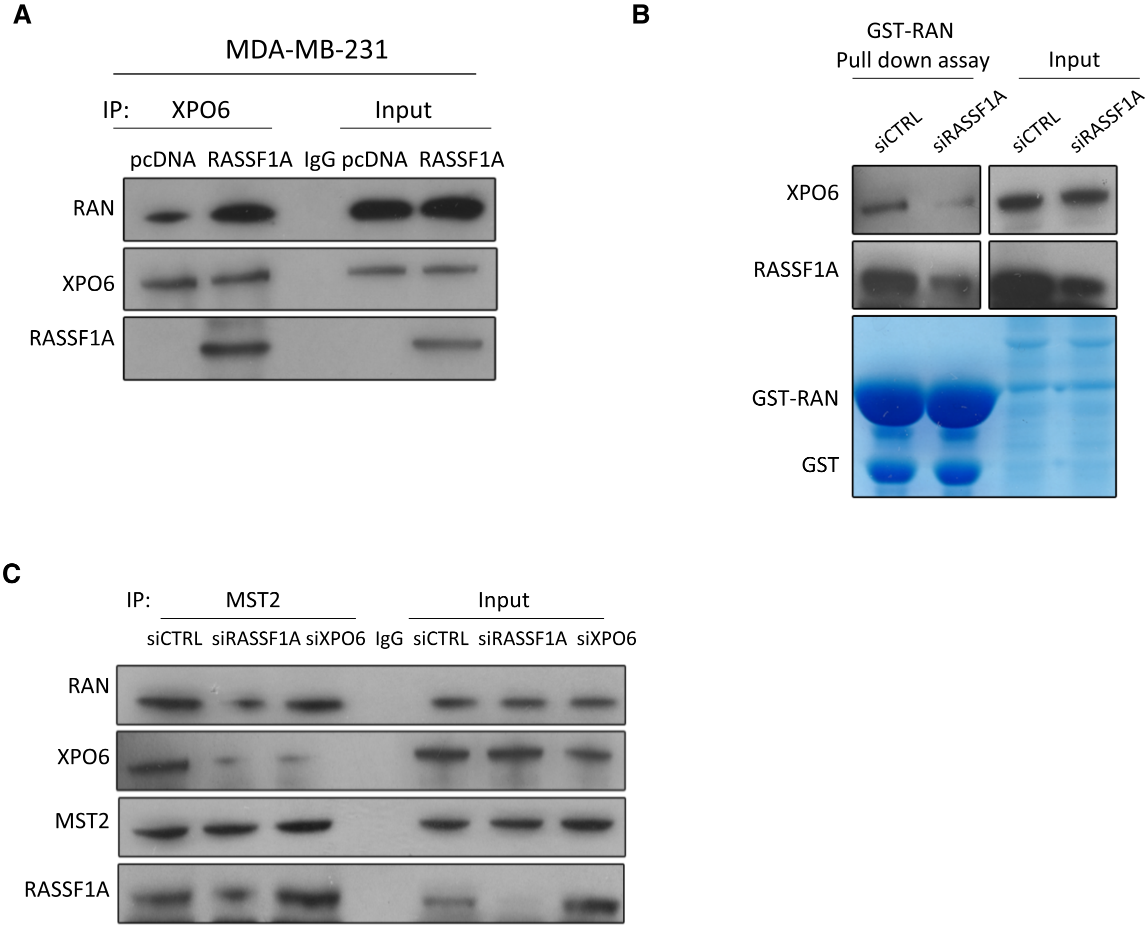

Figure EV3. RASSF1A interacts with XPO6 and RAN.

A Co-immunoprecipitation of endogenous XPO6 in MDA-MB-231 cells transfected with the empty vector pcDNA or with plasmid expressing RASSF1A.
B GST pull-down assays were performed using recombinant GST-RAN protein with siCTRL- or siRASSF1A-transfected HeLa total cell lysate.
C Co-immunoprecipitation of endogenous MST2 with endogenous XPO6, RAN and RASSF1A in siRNA-mediated knockdown of either RASSF1A or siXPO6 HeLa cells.

Source data are available online for this figure.

Figure EV4. RASSF1A depletion does not affect nuclear F-actin levels.

A Western blot analysis of actin and profilin levels in nuclear and cytoplasmic fractions of cells transfected with truncated RASSF1A mutants.
B Western blot analysis of actin and profilin levels in nuclear and cytoplasmic fractions of cells transfected with siRASSF1A and siRASSF1A together with XPO6 plasmid.
C Western blot analysis of actin and profilin levels in nuclear and cytoplasmic fractions of cells transfected with siRNA-resistant FLAG-RASSF1A plasmid.
D Western blot analysis of actin and profilin levels in nuclear and cytoplasmic fractions of cells transfected with siMST2. Quantification of nuclear actin and profilin relative to Lamin A/C is shown. Error bars derive from three independent experiments and represent the SEM.
E Western blot analysis of exportin-6 (XPO6) and importin-9 (IPO9) in the absence of RASSF1A. GAPDH was used as a loading control.
F Confocal images of endogenous filamentous actin (F-actin) in siCTRL and siRASSF1A cells using phalloidin staining (Alexa Fluor 568-conjugated, *red*). DNA was stained with DAPI. Scale bars = 10 μm.
G Western blot analysis of actin, profilin, GAPDH and Lamin A/C levels in nuclear and cytoplasmic fractions of MDA-MB-231 cells treated with DMSO or 5′-aza-dC. The graph shows the nuclear levels of actin and profilin relative to Lamin A/C in MDA-MB-231 cells expressing RASSF1A. Error bars derive from two independent experiments and represent the SEM.

Data information: Two-tailed Student's *t*-test was used for statistical analysis.*$P < 0.05$, **$P < 0.01$, ***$P < 0.001$.

Source data are available online for this figure.

© 2019 The Authors

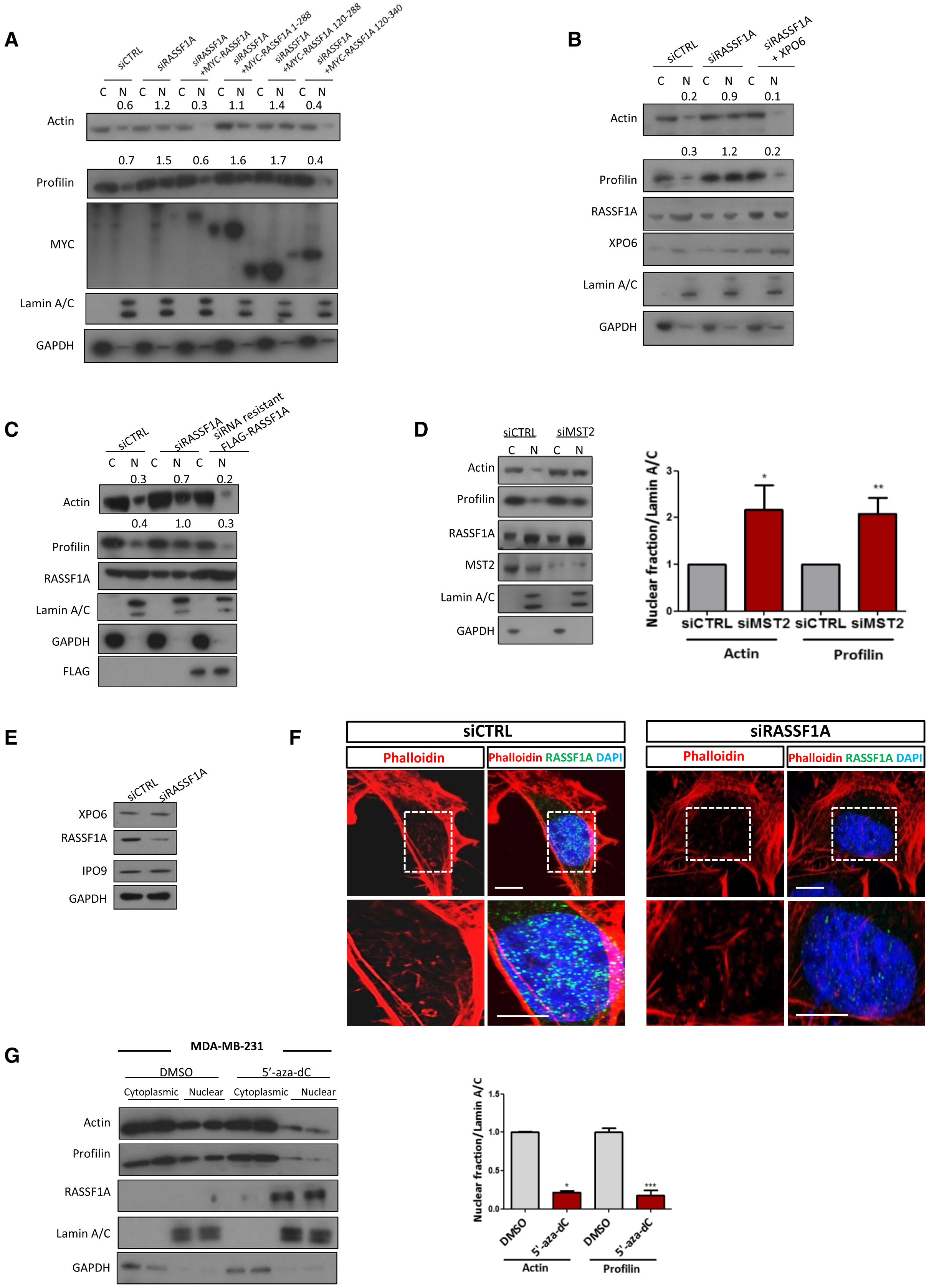

Figure EV4.

 © 2019 The Authors

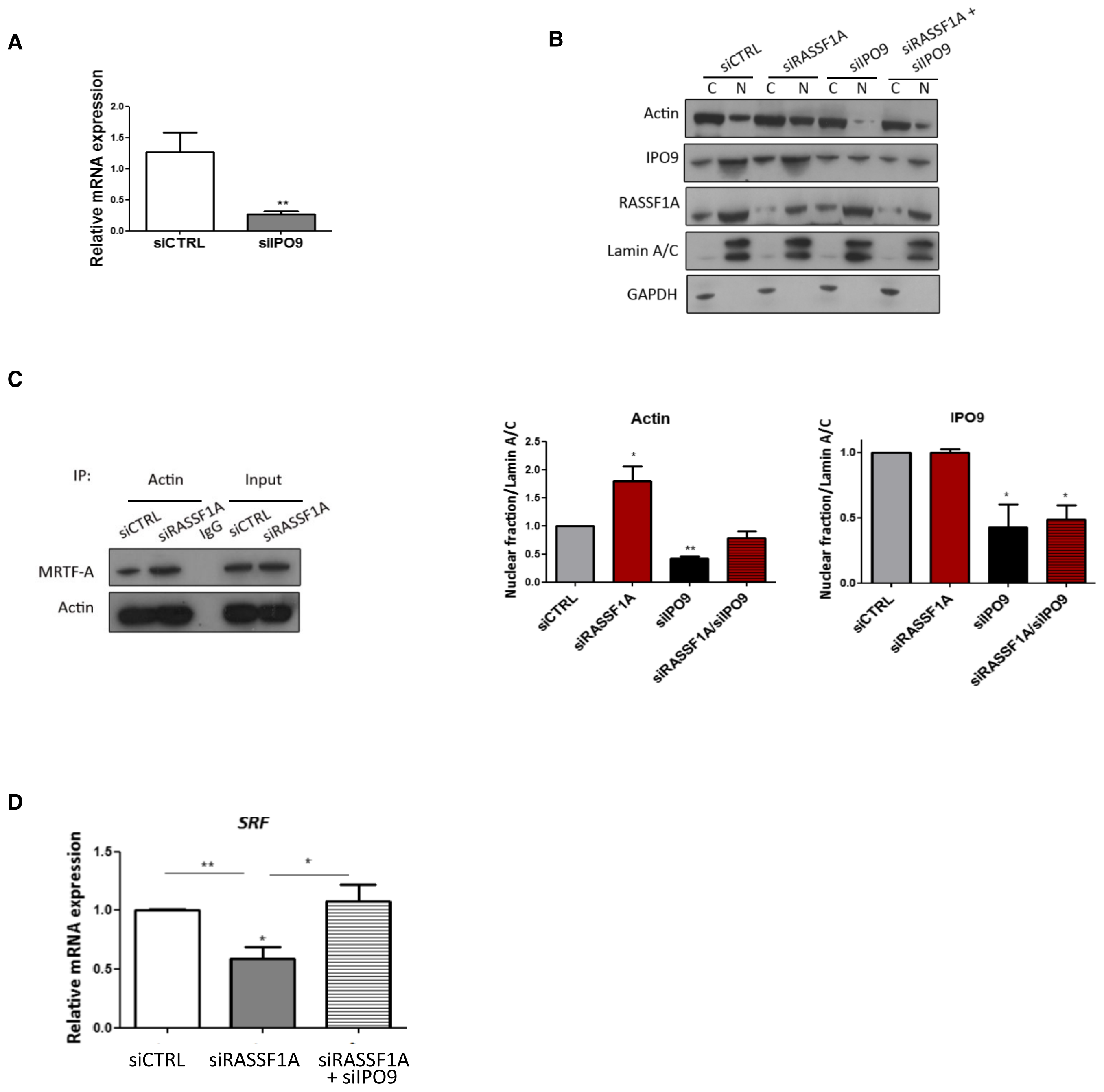

Figure EV5. Silencing of IPO9 restores RASSF1A-depleted effects on actin.

- A IPO9 mRNA levels in siIPO9-transfected HeLa cells relative to GAPDH and normalised to control siRNA levels. Data represent the mean ± SEM of three independent experiments.
- B Western blot analysis of actin protein levels following RASSF1A, IPO9 or RASSF1A/IPO9 knockdown in HeLa cells. Quantification of nuclear actin and IPO9 relative to Lamin A/C is shown. Error bars derive from three independent experiments and represent the SEM.
- C Co-immunoprecipitation of endogenous actin with endogenous MRTF-A in siRASSF1A-treated HeLa cells.
- D SRF mRNA levels in siRASSF1A or siRASSF1A + siIPO9-transfected HeLa cells relative to GAPDH and normalised to control siRNA levels. Data represent the mean ± SEM of three independent experiments.

Data information: Two-tailed Student's *t*-test was used for statistical analysis.*$P < 0.05$, **$P < 0.01$.

Source data are available online for this figure.

© 2019 The Authors

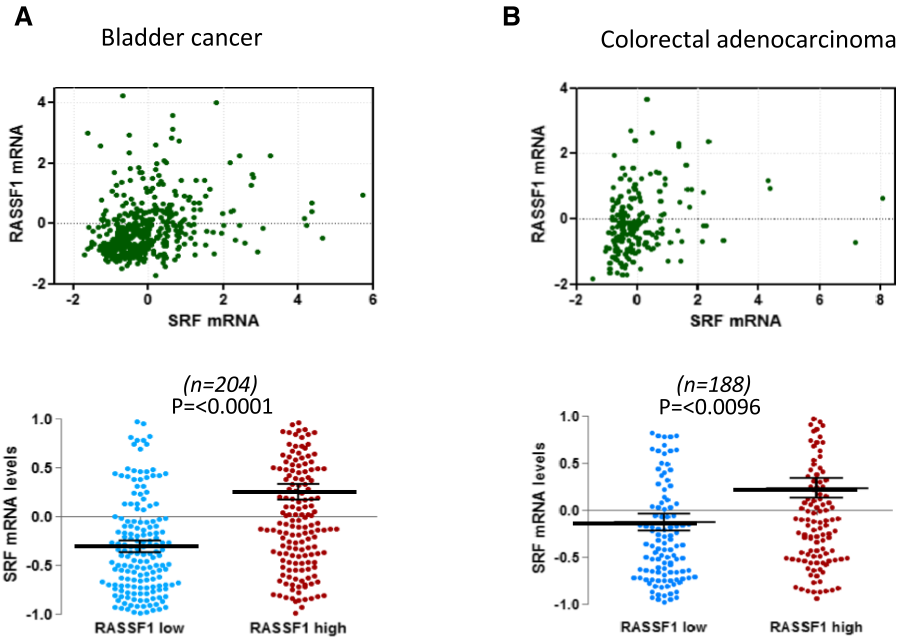

Figure EV6. Correlation of RASSF1/SRF transcripts in bladder and colorectal cancer.

A Upper: scatter plot of RASSF1 mRNA to SRF mRNA levels in bladder cancer (TCGA, Cell 2017, n = 404). Values are given in (RNA Seq V2 RSEM). Lower: differences in SRF mRNA expression of samples that express low and high levels of RASSF1 mRNA.

B Upper: scatter plot of RASSF1 mRNA to SRF mRNA levels in colorectal adenocarcinoma (TCGA, Nature 2012, n = 195). Values are given in (RNA Seq V2 RSEM). Lower: differences in SRF mRNA expression of samples that express low and high levels of RASSF1 mRNA.

 © 2019 The Authors
