# Supplementary figures and images for: RASSF1A is required for the maintenance of nuclear actin levels
Source: EMBO J. 2019 Jun 7;38(16):e101168. doi: 10.15252/embj.2018101168 (PMC6694222; doi:10.15252/embj.2018101168)

**Figure EV1A**

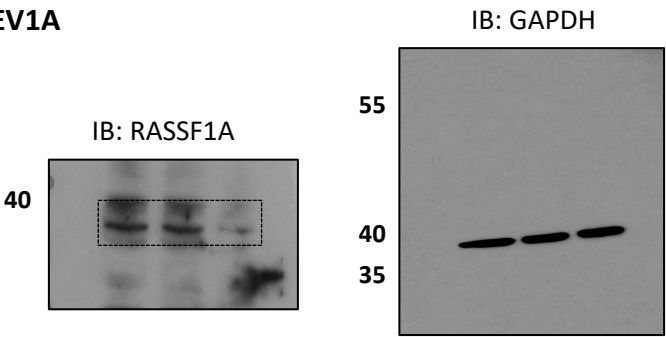

**Figure EV1C**

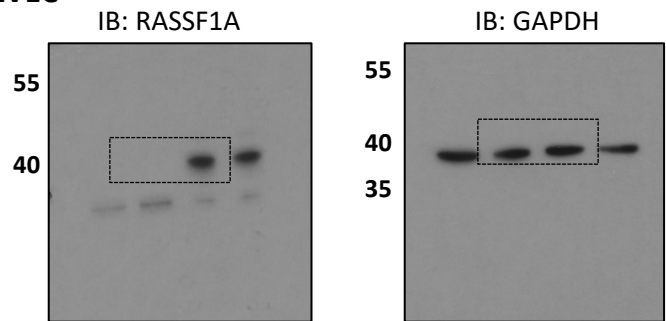

**Figure EV1F**

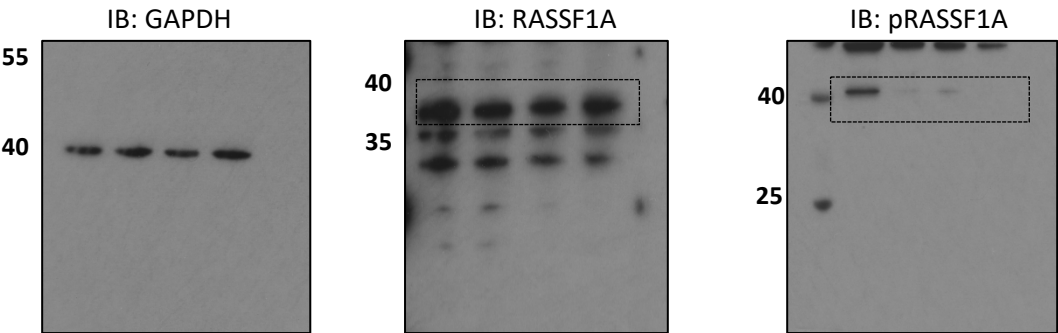

Supplement: Supplementary file 2 — Source Data for Expanded View [file EMBJ-38-e101168-s005.zip › Source_Data_EV_Figures/Source_Data_EV_Figures/SD_EV1.pdf]

Figure EV2A

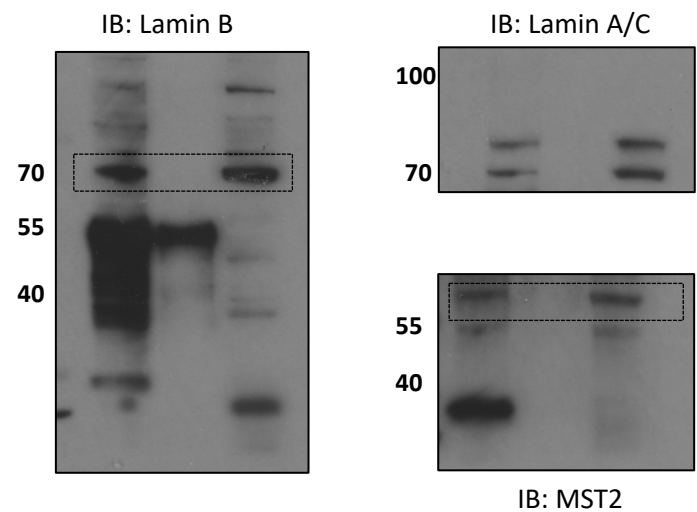

Figure EV2C

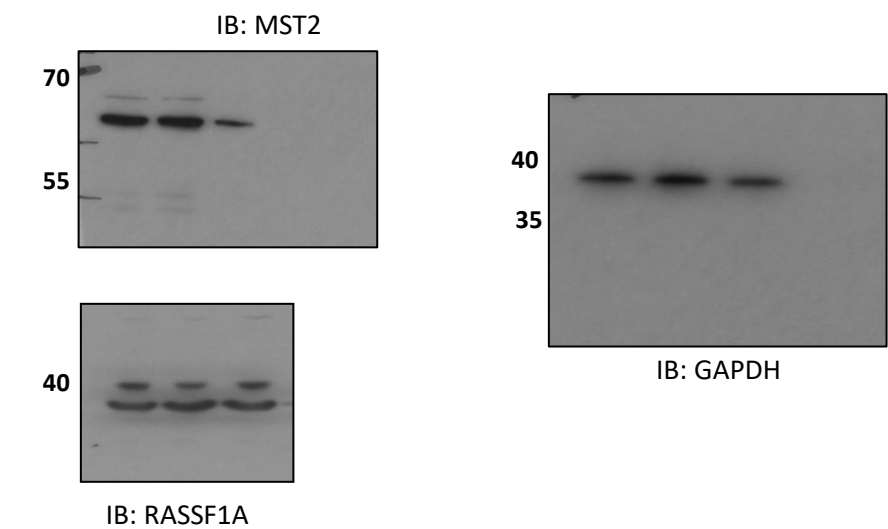

Supplement: Supplementary file 2 — Source Data for Expanded View [file EMBJ-38-e101168-s005.zip › Source_Data_EV_Figures/Source_Data_EV_Figures/SD_Fig_EV2.pdf]

Figure EV3A

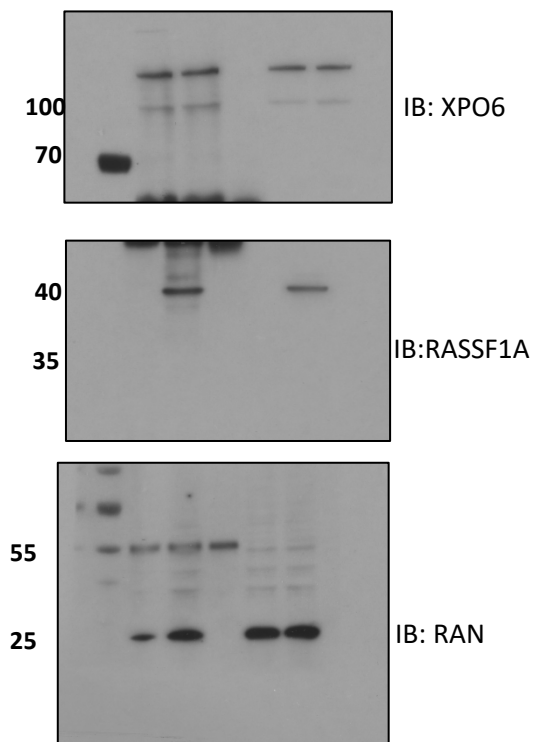

Figure EV3B

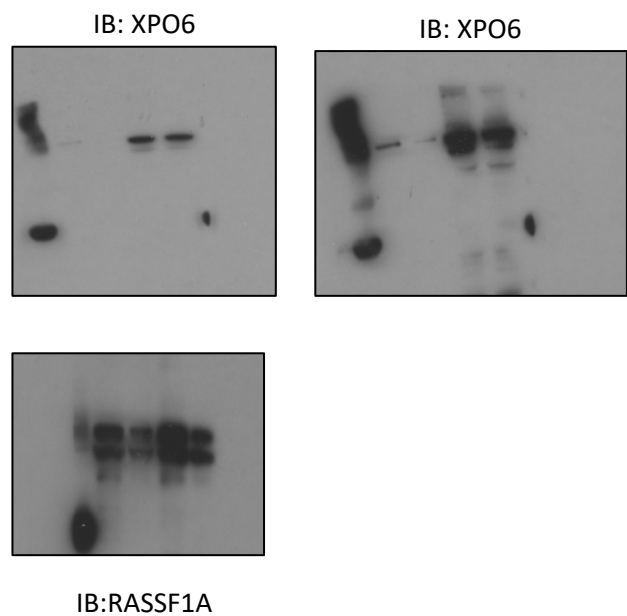

Figure EV3C

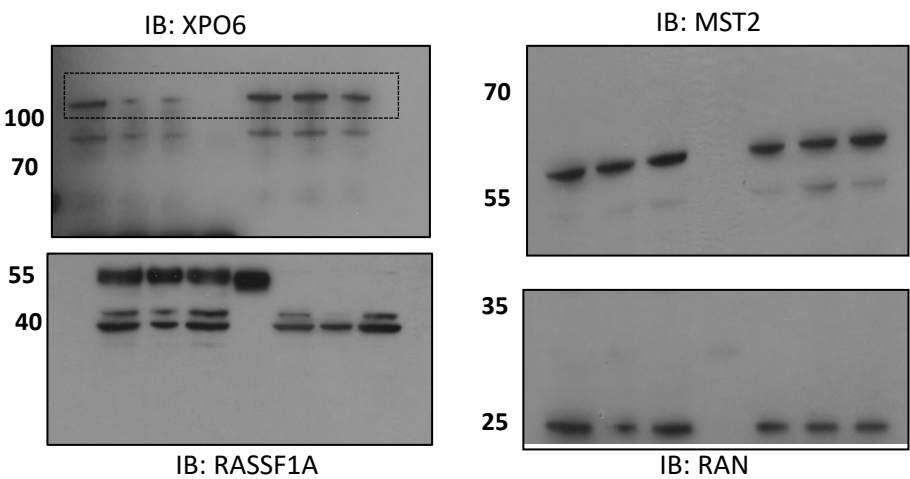

Supplement: Supplementary file 2 — Source Data for Expanded View [file EMBJ-38-e101168-s005.zip › Source_Data_EV_Figures/Source_Data_EV_Figures/SD_Fig_EV3.pdf]

Figure EV4A

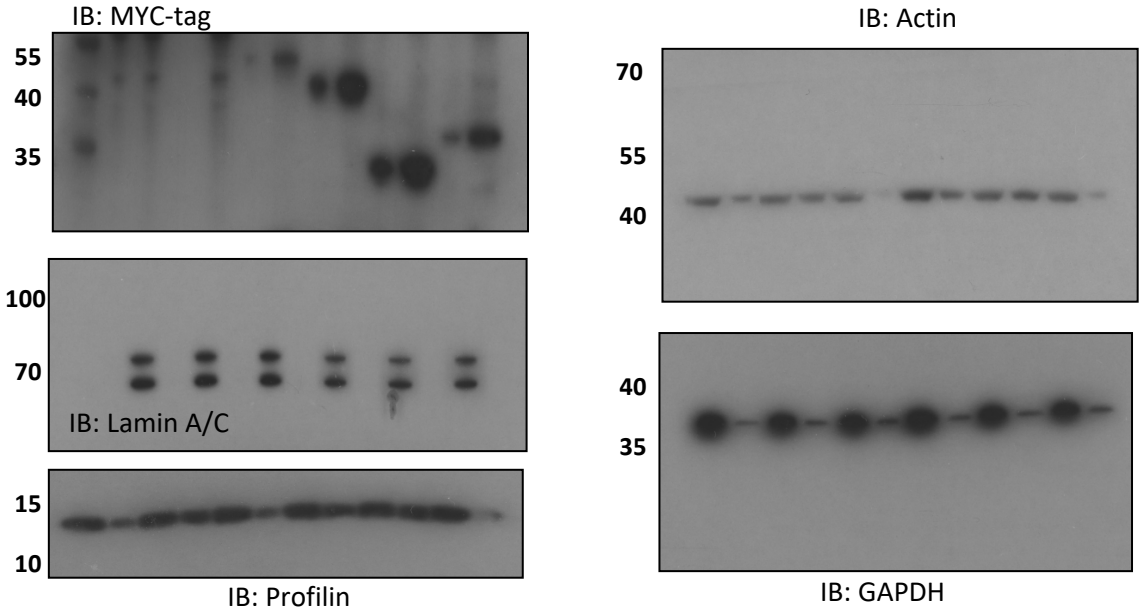

Figure EV4B

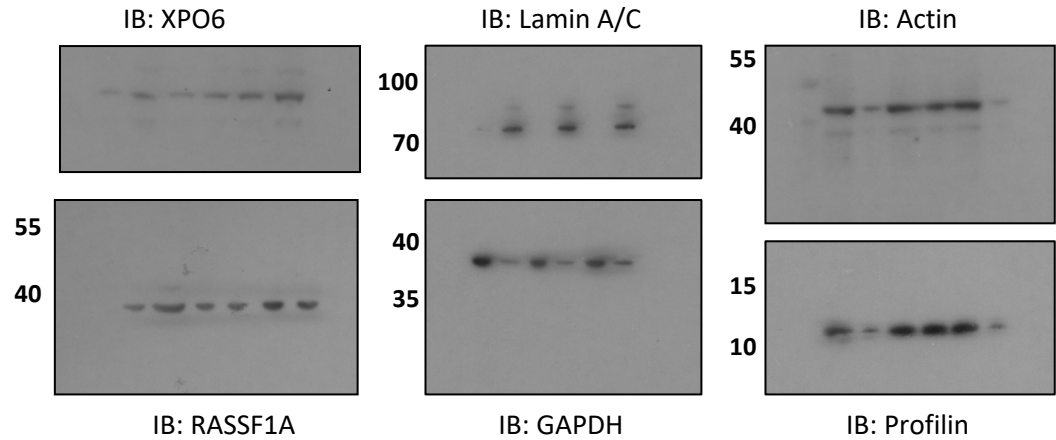

Figure EV4C

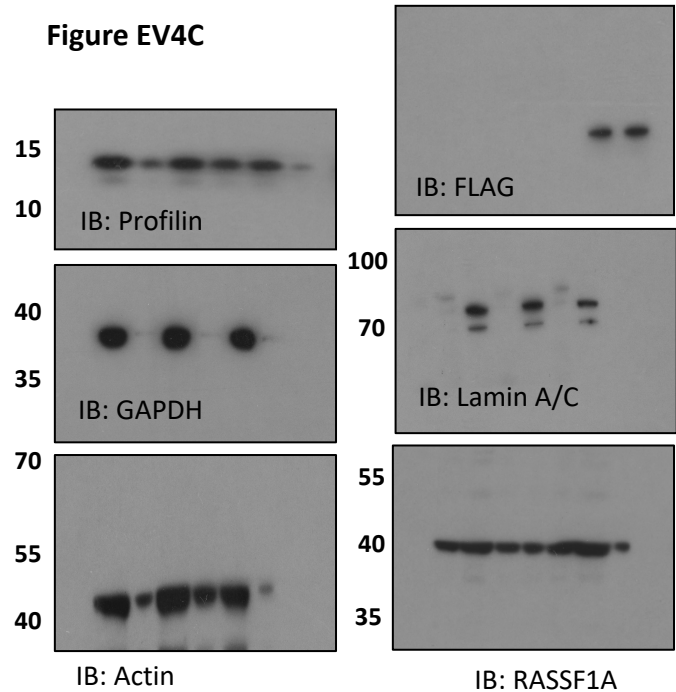

Supplement: Supplementary file 2 — Source Data for Expanded View [file EMBJ-38-e101168-s005.zip › Source_Data_EV_Figures/Source_Data_EV_Figures/SD_Fig_EV4_1.pdf]

Figure EV4D

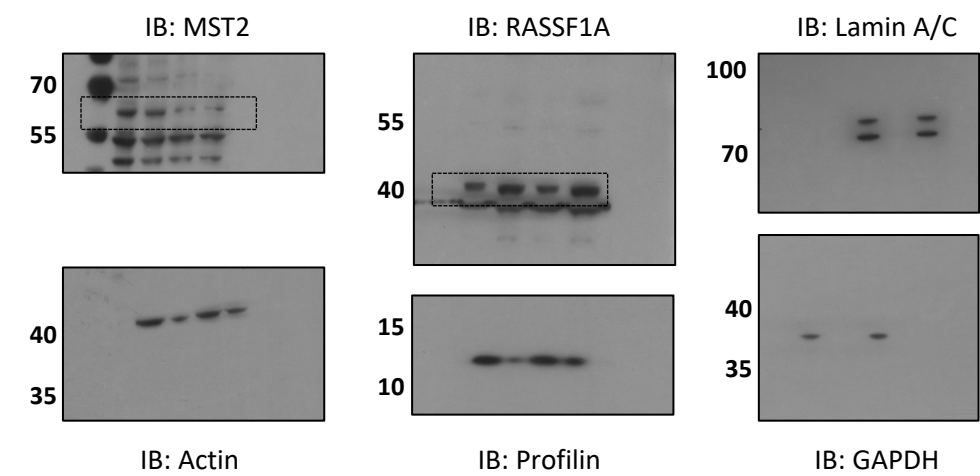

Figure EV4E

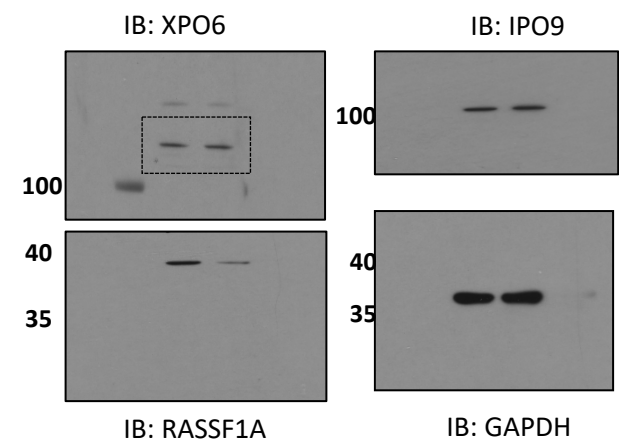

Figure EV4G

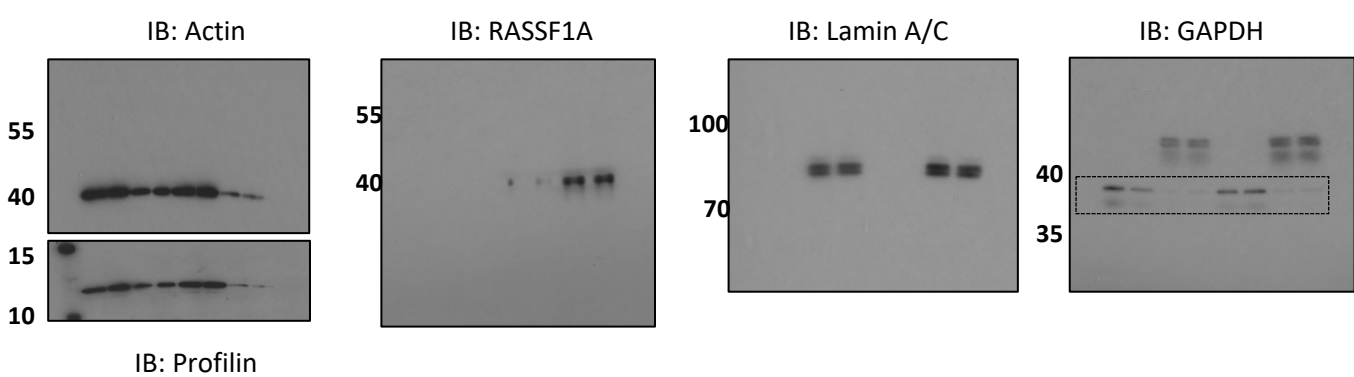

Supplement: Supplementary file 2 — Source Data for Expanded View [file EMBJ-38-e101168-s005.zip › Source_Data_EV_Figures/Source_Data_EV_Figures/SD_Fig_EV4_2.pdf]

Figure EV5B

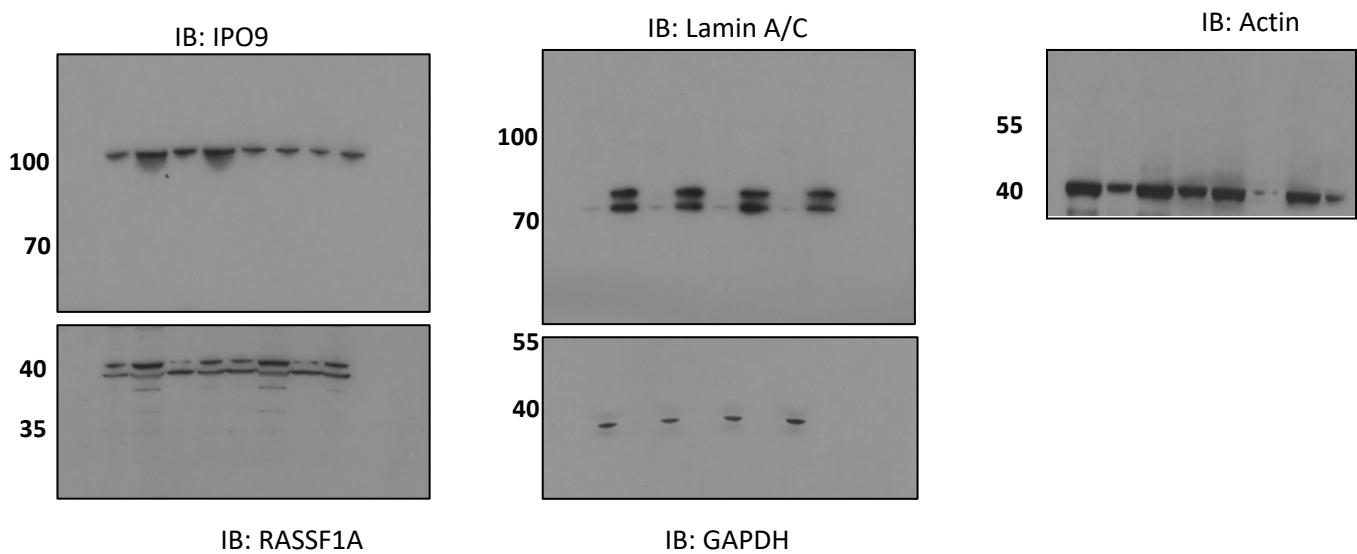

Figure EV5C

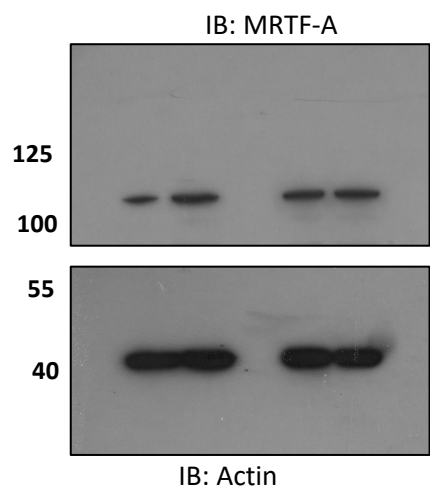

Supplement: Supplementary file 2 — Source Data for Expanded View [file EMBJ-38-e101168-s005.zip › Source_Data_EV_Figures/Source_Data_EV_Figures/SD_Fig_EV5.pdf]

Figure 1C

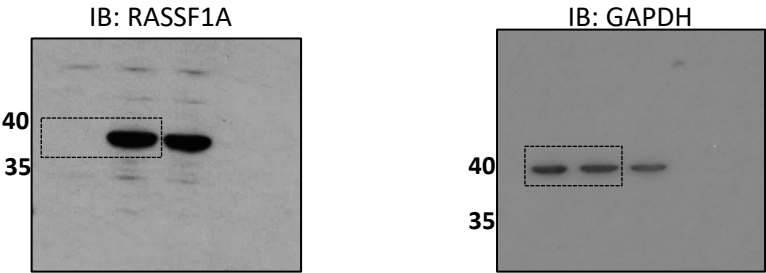

Supplement: Supplementary file 4 — Source Data for Figure 1 [file EMBJ-38-e101168-s002.pdf]

**Figure 3A**

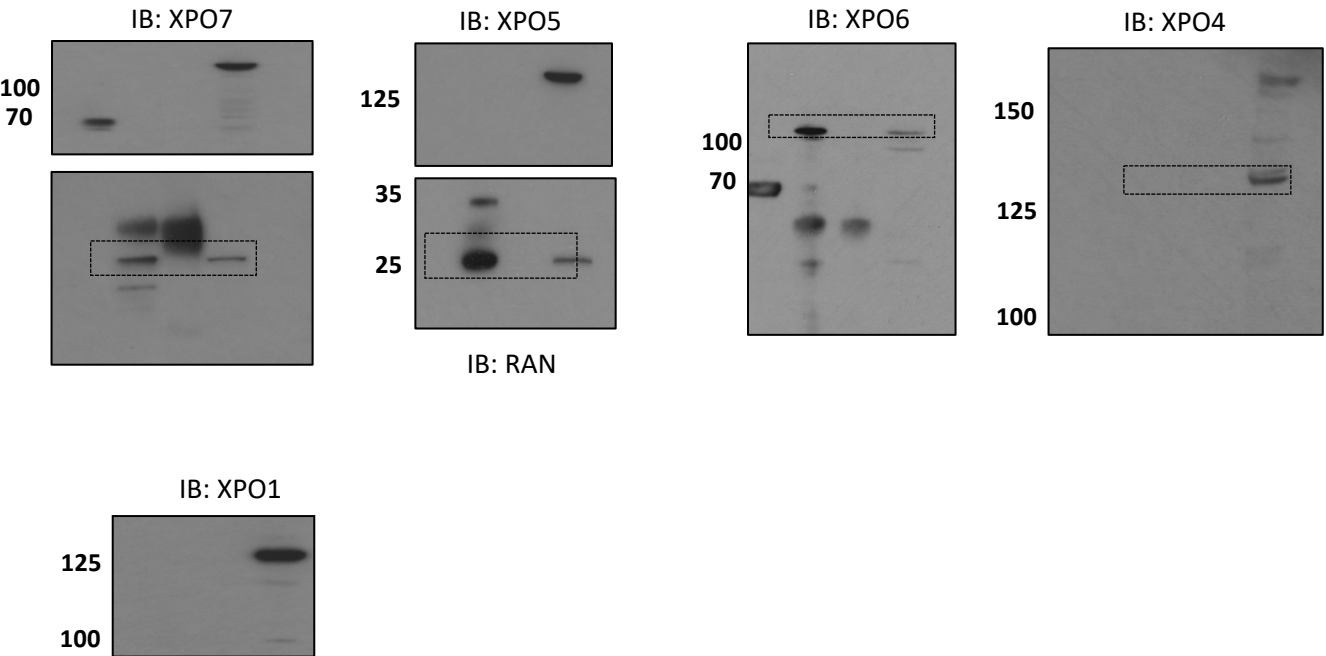

**Figure 3B**

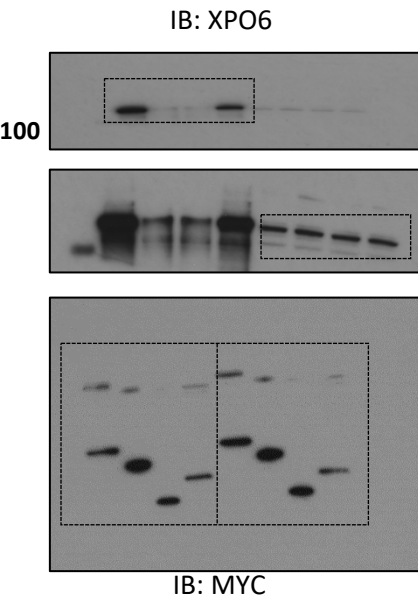

**Figure 3C**

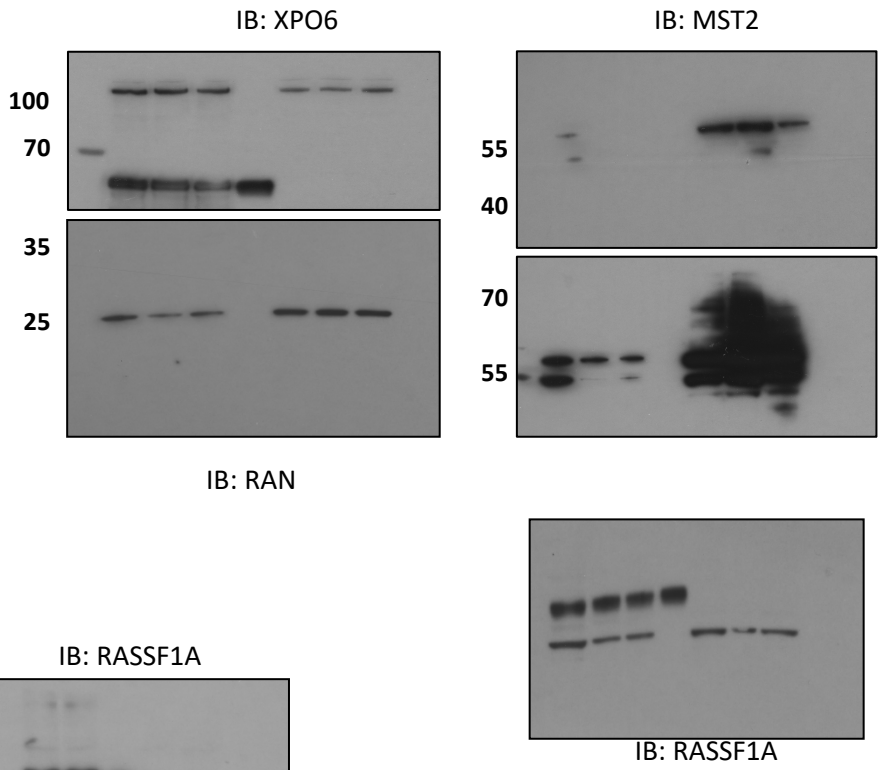

**Figure 3D**

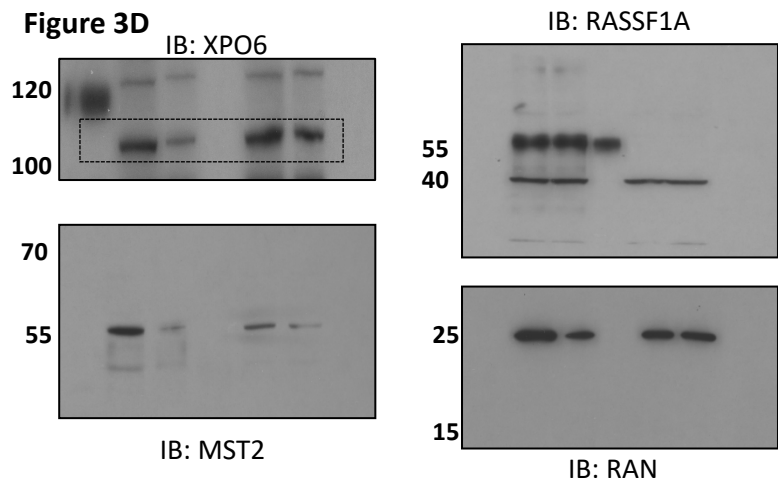

Supplement: Supplementary file 5 — Source Data for Figure 3 [file EMBJ-38-e101168-s003.pdf]

Figure 4A

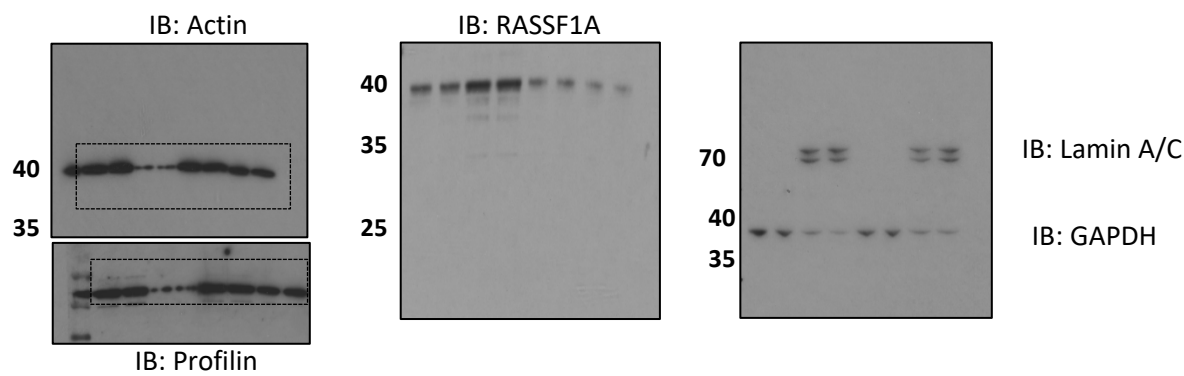

Figure 4D

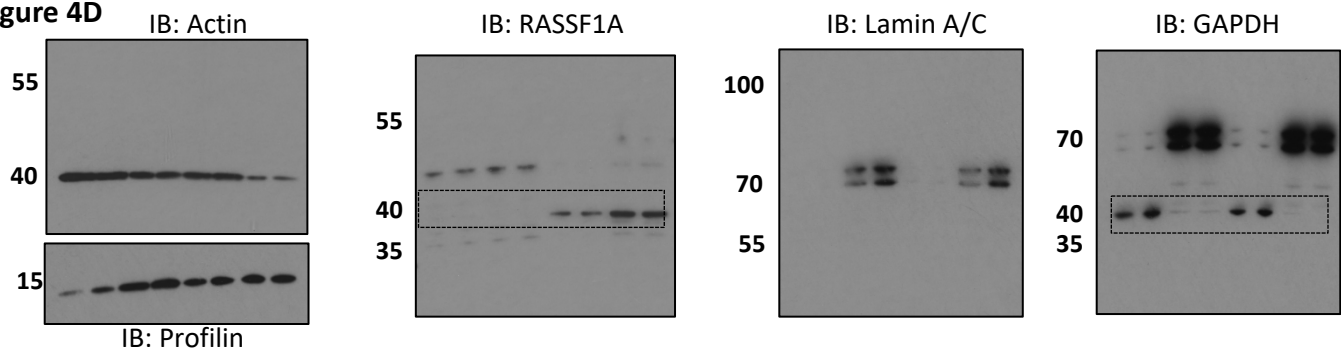

Supplement: Supplementary file 6 — Source Data for Figure 4 [file EMBJ-38-e101168-s004.pdf]
